# Measurement of mitochondrial respiratory chain enzymatic activities in *Drosophila melanogaster* samples

**DOI:** 10.1016/j.xpro.2022.101322

**Published:** 2022-04-15

**Authors:** Michele Brischigliaro, Elena Frigo, Erika Fernandez-Vizarra, Paolo Bernardi, Carlo Viscomi

**Affiliations:** 1 Department of Biomedical Sciences, University of Padova, Padova, Italy; 2 Veneto Institute of Molecular Medicine, Padova, Italy

**Keywords:** Cell separation/fractionation, Metabolism, Model Organisms, Protein Biochemistry

## Abstract

Mitochondrial respiratory chain (MRC) dysfunction is linked to mitochondrial disease as well as other common conditions such as diabetes, neurodegeneration, cancer, and aging. Thus, the evaluation of MRC enzymatic activities is fundamental for diagnostics and research purposes on experimental models. Here, we provide a verified and reliable protocol for mitochondria isolation from various *D. melanogaster* samples and subsequent measurement of the activity of MRC complexes I–V plus citrate synthase (CS) through UV-VIS spectrophotometry.

For complete details on the use and execution of this protocol, please refer to Brischigliaro et al. (2021).

## Highlights

• A detailed and quick protocol to isolate mitochondria from *D. melanogaster* samples
• A step-by-step procedure to measure MRC enzymatic activities in isolated mitochondria
• A comprehensive guide for data analysis, with examples of validated systems

## Graphical abstract

**Figure:**
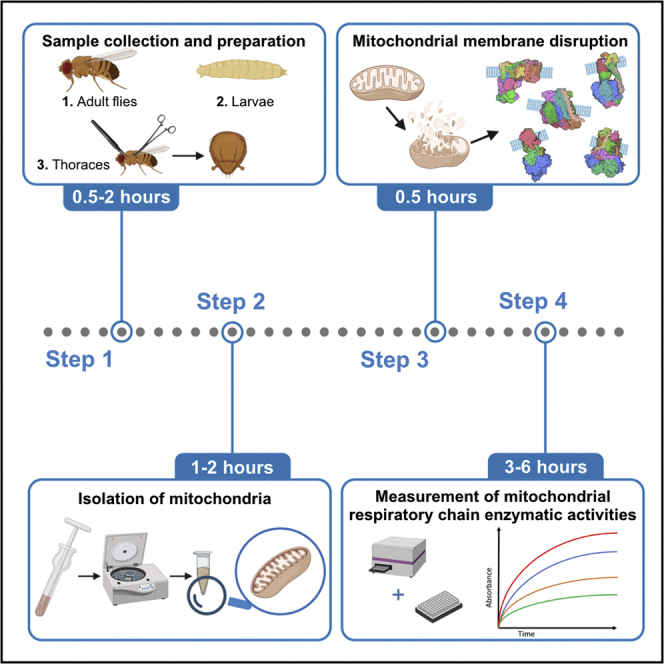

## Before you begin

This protocol describes step-by-step how to measure the enzymatic activity of the MRC complexes in isolated mitochondria obtained from different *Drosophila melanogaster* tissues. Firstly, mitochondria are separated from homogenized tissues through differential centrifugation using a sucrose-based buffer. Then, mitochondria membranes are disrupted to allow the substrates to access the MRC complexes. Enzymatic activities of individual MRC complexes are measured using specific kinetic assays and UV-VIS spectrophotometry. The MRC activities measured in this way are often normalized to the activity of the citrate synthase, a mitochondrial matrix enzyme of the Krebs cycle that is considered as a good marker of mitochondrial volume. This detailed protocol builds on previously described methods for the isolation of mitochondria from *D. melanogaster* tissues (Brischigliaro et al., 2019; Brischigliaro et al., 2021) and on the procedures for measuring MRC enzymatic activities in human samples (Bugiani et al., 2004; Kirby et al., 2007; Spinazzi et al., 2012; Trounce et al., 1996). However, extensive optimization was carried out by the authors in order to provide a detailed and step-by-step procedure easy to follow also for newcomers.

Essential material you must have/prepare:

1. Biological samples: adult files or larvae.
2. Refer to key resources table and materials and equipment to prepare reagents and buffers.

### Sample collection and preparation

**Timing: 0.5–2 h**

MRC enzymatic activities can be measured in different *Drosophila* samples, i.e., whole adult flies, larvae, or adult thoraces.

Sample collection and preparation:

3. Adult flies: collect 80–100 adult flies per biological replicate.
4. Larvae: collect 80–100 third instar larvae per biological replicate. To pick up larvae, add tap water supplemented with sugar (20%) into the vials where larvae are growing and let them detach from the food or from the tube walls; when they are floating, take and wash them with PBS using a strainer.
5. Adult thoraces: collect 120–150 thoraces per biological replicate. For dissection, anesthetize flies with CO_2_, quickly rinse them in 70% ethanol to remove cuticle wax. Dissect thoraces in ice-cold PBS using tweezers and precision scissors by removing the head, the abdomen, the wings and the legs.

***Note:*** The number of larvae and thoraces should be adjusted based on their size and experimental needs. Generally, 80–100 wild-type third instar larvae or 120–150 wild-type thoraces are needed to obtain approximately 200–400 μg of isolated mitochondrial protein.

***Note:*** This protocol can be applied to virtually any biological sample. The amount of sample must be empirically determined. Generally, 20–30 mg of tissue are needed.

## Key resources table

**Table:** 

REAGENT or RESOURCE	SOURCE	IDENTIFIER
**Chemicals, Peptides, and Recombinant Proteins**		

Acetyl coenzyme A lithium salt	Merck	A2181
Adenosine 5′-triphosphate disodium salt hydrate	Merck	A2383
Antimycin A	Merck	A8674
BSA (fatty acid free)	Merck	A6003
Coenzyme Q_1_	Merck	C7956
Cytochrome *c* from equine heart	Merck	C7752
D-Mannitol	Merck	M4125
DCIP (2,6-Dichloroindophenol Sodium Salt Hydrate)	Merck	D1878
Decylubiquinone	Merck	D7911
DTNB (5,5′-Dithiobis(2-nitrobenzoic acid))	Merck	D218200
EDTA (Ethylenediaminetetraacetic acid disodium salt dihydrate)	Merck	E1644
EGTA (Ethylene glycol-bis(2-aminoethylether)-N,N,N′,N′-tetraacetic acid)	Merck	E3889
HEPES (N-(2-Hydroxyethyl)piperazine-N′-(2-ethanesulfonic acid), 4-(2-Hydroxyethyl)piperazine-1-ethanesulfonic acid)	Merck	H3375
KCN (Potassium cyanide)	Merck	31252
L-Lactic Dehydrogenase from rabbit muscle	Merck	L2500
Magnesium chloride hexahydrate	Merck	M2670
Malonic acid	Merck	M1296
NADH (β-Nicotinamide adenine dinucleotide, reduced dipotassium salt)	Merck	N4505
Oligomycin from Streptomyces diastatochromogenes	Merck	O4876
Oxaloacetic acid	Merck	O4126
Phospho(enol)pyruvic acid monopotassium salt	Merck	860077
Potassium borohydride	Merck	438472
Potassium phosphate dibasic	Merck	P2222
Potassium phosphate monobasic	Merck	P5655
Pyruvate Kinase from rabbit muscle	Merck	P1506
Rotenone	Merck	R8875
Sodium hydrosulphite	Merck	157953
Sodium succinate	Merck	S2378
Sucrose	Merck	S7903
TRIS base	Merck	T1503
Tween-20	Merck	P7949

**Experimental models: Organisms/strains**		

*D. melanogaster* strain expressing GAL4 under the control of the Act5c promoter. y[1] w[∗]; P{w[+mC]=Act5C-GAL4}25FO1/CyO, y[+]	Bloomington Drosophila Stock Center	BDSC 4414
*D. melanogaster* strain expressing a transgenic RNAi construct for *ND-18*	Vienna Drosophila Resource Center	VDRC 101489
*D. melanogaster* strain expressing a transgenic RNAi construct for *Bcs1*	Bloomington Drosophila Stock Center	BDSC 51863
*D. melanogaster* strain expressing a transgenic RNAi construct for *Coa8*	Vienna Drosophila Resource Center	VDRC 100605

**Software and algorithms**		

Excel	Microsoft	Office suite

**Other**		

96-well clear flat bottom plates	Corning	3628
Thermo mixer	Eppendorf	5382000015
Tissue grinder	VWR	432-0202
UV-VIS Spectrophotometer plate reader	Thermo Fisher Scientific	VL0LA0D0
Stirrer motor	IKA	3593000
Centrifuge Tubes - 30 mL	Thermo Fisher Scientific	3119-0030
Cell strainers	Corning	431752
Benchtop dewar flask	Thermo Fisher Scientific	4150-1000

## Materials and equipment

### Buffers for isolation of mitochondria and sample preparation

• **MS (mannitol-sucrose) buffer:**

**Table:** 

Reagent	Final concentration
Mannitol	225 mM
Sucrose	75 mM
HEPES-KOH pH 7.4	5 mM
EGTA	1 mM
ddH_2_O	n/a
**Total**	**200 mL**

Store at 4°C for 1 week.

• **MS buffer + BSA 1%:** dissolve 1.5 g of fatty acid free BSA in 150 mL of MS buffer. Store at 4°C for 1 week.
• **Tris-HCl (10 mM pH 7.4):** dissolve 61 mg of Tris base in 15 mL of distilled H_2_O, adjust pH to 7.4, adjust the volume to 50 mL with distilled H_2_O. Store at 4°C for 1 month.

### Reaction buffers

• **Potassium phosphate monobasic (0.5 M):** dissolve 6.8 g of potassium phosphate monobasic (KH_2_PO_4_) in 80 mL of distilled H_2_O, adjust the volume to 100 mL. Store at 4°C for 1 month.
• **Potassium phosphate dibasic (0.5 M):** dissolve 8.71 g of potassium phosphate dibasic (K_2_HPO_4_) in 80 mL of distilled H_2_O, adjust the volume to 100 mL. Store at 4°C for 1 month.
• **Potassium phosphate monobasic (0.1 M):** dilute 10 mL of 0.5 M potassium phosphate monobasic in 40 mL of distilled H_2_O. Store at 4°C for 1 month.
• **Potassium phosphate dibasic (0.1 M):** dilute 10 mL of 0.5 M potassium phosphate dibasic in 40 mL of distilled H_2_O. Store at 4°C for 1 month.
• **Potassium phosphate (0.5 M pH 7.5):** adjust 50 mL of 0.5 M potassium phosphate dibasic to pH 7.5 with 0.5 M potassium phosphate monobasic. Store at 4°C for 1 month.
• **Potassium phosphate (0.1 M pH 7.0):** adjust 50 mL of 0.1 M potassium phosphate dibasic to pH 7.0 with 0.1 M potassium phosphate monobasic. Store at 4°C for 1 month.
• **Tris-HCl + Triton X-100:**

**Table:** 

Reagent	Final concentration
Tris-HCl pH 8.0	0.2 M
Triton X-100	0.2%
ddH_2_O	n/a
**Total**	**100 mL**

Store at 4°C for 1 month.

• **HEPES-KOH + MgCl**_**2**_ **:**

**Table:** 

Reagent	Final concentration
HEPES-KOH pH 8.0	0.1 M
MgCl_2_	10 mM
ddH_2_O	n/a
**Total**	**100 mL**

Store at 4°C for 1 month.

### Reagents for complex I (NADH:ubiquinone oxidoreductase) assay

• **BSA (50 mg/mL):** dissolve 50 mg of fatty-acid free BSA in 1 mL of distilled H_2_O. Store at 4°C for 2 days or at −20°C for 1 month.
• **KCN (10 mM):** dissolve 3.3 mg of potassium cyanide (KCN) in 5 mL of distilled H_2_O. Prepare fresh before the assay.

**CRITICAL:** KCN is highly toxic, under acidic conditions volatile and extremely toxic HCN species form. Handle KCN and KCN solution under a fume hood using PPE.

***Note:*** 50 mM sodium azide (NaN3) can be used as an alternative.

• **NADH (5 mM):** dissolve 3.7 mg of β-Nicotinamide adenine dinucleotide (NADH) in 1 mL of distilled H_2_O. Prepare fresh before the assay.
• **Coenzyme Q**_**1**_**(4 mM):** dissolve 2 mg of Coenzyme Q_1_ (CoQ_1_) in 2 mL of 100% ethanol. Prepare 100- μL aliquots and store at −20°C for 6 months.
• **Rotenone (1 mM):** dissolve 3.9 mg of rotenone in 10 mL of 100% ethanol. Prepare 1-mL aliquots and store and −20°C for 6 months.
  **CRITICAL:** rotenone is highly toxic, handle it under a fume hood using PPE.

### Reagents for complex II (succinate:ubiquinone oxidoreductase) assay

• **Succinate (400 mM):** dissolve 2.36 g of sodium succinate in 20 mL of distilled H_2_O, adjust the pH to 7.4 using NaOH, adjust the volume to 50 mL with distilled water. Store in 2-mL aliquots at −20°C for 6 months.
• **DCPIP (500 μM):** dissolve 2.9 mg of 2,6-dichlorophenolindophenol (DCPIP) in 20 mL of distilled H_2_O. Protect from light. Store at −20°C for 1 month.
• **KCN (10 mM):** dissolve 3.3 mg of potassium cyanide (KCN) in 5 mL of distilled H_2_O. Prepare fresh before the assay.

**CRITICAL:** KCN is highly toxic, under acidic conditions volatile and extremely toxic HCN species form. Handle KCN and KCN solution under a fume hood using PPE.

***Note:*** 50 mM sodium azide (NaN3) can be used as an alternative.

• **Coenzyme Q**_**1**_**(4 mM):** dissolve 2 mg of Coenzyme Q_1_ in 2 mL of 100% ethanol. Prepare 100- μL aliquots and store at −20°C for 6 months.
• **Malonate (1 M):** dissolve 208 mg of malonic acid in 2 mL of distilled H_2_O. Store at −20°C for 6 months.

### Reagents for complex III (ubiquinol:cytochrome c oxidoreductase) assay

• **EDTA (5 mM, pH 7.5):** dissolve 93 mg of EDTA (ethylenediaminetetraacetic acid) in 40 mL of distilled H_2_O, adjust pH to 7.5 with NaOH, adjust the volume to 50 mL. Store at room temperature for 6 months.
• **Tween-20 (2.5%):** dissolve 125 μL of Tween-20 in 4.875 mL of distilled H_2_O. Protect from light. Prepare fresh before the assay.
• **Cytochrome *c* (1 mM, oxidized):** dissolve 12.4 mg in 1 mL of 20 mM potassium phosphate buffer, pH 7.0. Prepare fresh before the assay.
• **Decylubiquinol (10 mM):** dissolve 10 mg decylubiquinone in 3.1 mL of 100% ethanol. Store in 200-μL aliquots at −20°C for 6 months. To prepare decylubiquinol (reduced decylubiquinone), add few crystals of potassium borohydride to a 200-μL aliquot of decylubiquinone, add few (usually 2–4) 5 μL-aliquots of 0.1 M HCl, until the solution becomes colorless. Centrifuge at 10,000 × *g* for 1 min, transfer the solution to a new tube without carrying over borohydride crystals. Add few (usually 2–3) 5 μL-aliquots of 1 M HCl, until the solution stops bubbling. Keep on ice. Perform the reduction before the assay, do not store and reuse.
  **CRITICAL:** potassium borohydride is highly toxic, handle it under a fume hood using PPE.
• **KCN (10 mM):** dissolve 3.3 mg of potassium cyanide (KCN) in 5 mL of distilled H_2_O. Prepare fresh before the assay.

**CRITICAL:** KCN is highly toxic, under acidic conditions volatile and extremely toxic HCN species form. Handle KCN and KCN solution under a fume hood using PPE.

***Note:*** 50 mM sodium azide (NaN_3_) can be used as an alternative.

• **Antimycin A (1 mg/mL):** dissolve 4 mg of antimycin A in 4 mL of 100% ethanol. Store at −20°C for 6 months.
  **CRITICAL:** antimycin is highly toxic, handle it and dissolve under a fume hood using PPE.

### Reagents for complex IV (cytochrome c oxidase) assay

• **Cytochrome *c* (1 mM, reduced):** dissolve 12.4 mg in 1 mL of 20 mM potassium phosphate buffer, pH 7.0. Add few crystals of sodium dithionite and mix, until the color of the solution switches from dark brown to orange/pink. Prepare a 20 μM solution and measure the absorbance at 550 nm and 565 nm, calculate the ratio A550/A565. Optimal values are between 6 and 8. If the ratio is lower than 6 repeat the procedure. Prepare fresh before the assay.
• **KCN (10 mM):** dissolve 3.3 mg of potassium cyanide (KCN) in 5 mL of distilled H_2_O. Prepare fresh before the assay.

**CRITICAL:** KCN is highly toxic, under acidic conditions volatile and extremely toxic HCN species form. Handle KCN and KCN solution under a fume hood using PPE.

***Note:*** 50 mM sodium azide (NaN3) can be used as an alternative.

### Reagents for citrate synthase assay

• **DTNB (1 mM):** dissolve 4 mg of 5,5′-dithiobis (2-nitrobenzoic acid), (DTNB) in 10 mL of 100 mM Tris-HCl pH 8.0. Protect from light. Prepare fresh before the assay.
• **Acetyl-CoA (10 mM):** dissolve 10 mg of Acetyl-Coenzyme A in 1.24 mL of distilled H_2_O. Prepare 100 μL-aliquots and store at −80°C for 6 months.
• **Oxaloacetate (10 mM):** dissolve 2.6 mg of oxaloacetic acid in 2 mL of distilled H_2_O. Prepare fresh before the assay.

### Reagents for complex V (ATP synthase) assay

• **Phosphoenolpyruvate (50 mM):** dissolve 21 mg of phosphoenolpyruvic acid in 2 mL of distilled H_2_O. Store at −20°C for 6 months.
• **NADH (5 mM):** dissolve 3.7 mg of β-Nicotinamide adenine dinucleotide (NADH) in 1 mL of distilled H_2_O. Prepare fresh before the assay.
• **ATP (25 mM pH 7.0):** add 7 mL of distilled H_2_O to 138 mg of adenosine triphosphate (ATP), dissolve by adjusting pH to 7.0 with 2 M NaOH. Adjust the volume to 10 mL. Prepare 1 mL-aliquots and store at −80°C for 6 months.
• **Antimycin A (0.2 mg/mL):** dissolve 1 mg of antimycin A in 5 mL of 100% ethanol. Store at −20°C for 6 months.
  **CRITICAL:** antimycin is highly toxic, handle it and dissolve under a fume hood using PPE.
• **Oligomycin (5 mM):** dissolve 4 mg of oligomycin in 1 mL 100% ethanol. Prepare 200 μL-aliquots and store at −20°C for 6 months.

## Step-by-step method details

### Isolation of mitochondria

**Timing: 1–2 h**

Prior to measurement of mitochondrial respiratory chain enzymatic activities, mitochondria are prepared by differential centrifugation as described below.

**CRITICAL:** samples must always be kept on ice, even during the homogenization step, using ice-cold buffers to minimize protein degradation.

1. Homogenize samples in 2–3 mL of MS buffer with BSA, with 15–20 strokes at 1,000 rpm in a motor driven Teflon-glass Elvehjem potter on ice. Make sure the samples are always cold to avoid protein degradation.

***Note:*** if a motor driven potter is not available, use a Dounce glass potter with a loose-fitting glass pestle for adults and third instar larvae and a tight-fitting glass pestle for thoraces and small larvae.

**CRITICAL:** during the homogenization step, apply the same number of strokes to all the samples to obtain consistent results.

2. Transfer the homogenate to a clean 30 mL centrifuge tube, rinse the potter with 7 mL of MS buffer with BSA and transfer to the tube with homogenate.
3. Centrifuge the homogenate at 1,000 × *g* for 10 min at 4°C, to separate the liquid phase from debris, i.e., unbroken cells and nuclei.

**CRITICAL:** centrifuge steps must be performed at 4°C to minimize sample degradation.

4. Filter supernatant through a 100 μm strainer and transfer it into a new tube.

***Note:*** filtering step is needed to remove legs, wings and cuticle debris that tend to float after homogenization.

5. Centrifuge samples at 6,000 × *g* for 10 min at 4°C to pellet the mitochondrial fraction.

**CRITICAL:** After the centrifugation steps, lipids are present as a white layer adhering to the tube walls. Make sure to remove lipids during the washing steps to avoid interference during measurement of enzymatic activities.

6. Discard supernatant and resuspend the pellet with 10 mL of MS buffer with BSA. Repeat step 5.
7. Remove the supernatant and resuspend the pellet with 10 mL of MS buffer without BSA.
8. Centrifuge samples at 7,000 × *g* for 10 min at 4°C.
9. Discard the supernatant and resuspend the pellet in 300–400 μL of MS buffer without BSA.
10. Measure protein concentration with method of choice (e.g., Bradford, Lowry, or BCA).

***Note:*** protein concentration should be measured in triplicate setting up a standard curve, to achieve good consistence and reliability of the results.

***Note:*** this procedure yields crude mitochondrial fractions. Samples can be stored at −80°C for maximum 3 months. Avoid multiple freeze and thaw cycles of pellets to minimize degradation of protein complexes that can result in artifactual reduction of enzyme activities. Isolated mitochondria preparations can be stored in aliquots to avoid multiple freeze and thaw cycles.

**Pause point:** Proceed with protocol or store samples at −80°C for up to 3 months.

### Disruption of mitochondrial membranes

**Timing: 0.5 h**

Prior to the activity assays, mitochondrial membranes need to be disrupted in order to allow access of the substrates to the MRC enzyme complexes.

11. Pellet isolated mitochondria by centrifuging samples at 7,000 × *g* for 5 min at 4°C.

***Note:*** depending on the type and the number of assays to be performed, evaluate the total amount of mitochondria needed for the experiment. For further details on the amounts of mitochondria needed for each assay, check the specific protocols below.

12. Carefully discard supernatant and resuspend the pellet with hypotonic buffer (10 mM Tris-HCl pH 7.6) at 1 μg/μL final protein concentration.
13. Snap-freeze in liquid nitrogen. Thaw at 25°C in a benchtop thermo shaker or, alternatively, in a water bath. Perform three freeze-thaw cycles to enhance mitochondrial membrane disruption.

***Note:*** At this point, mitochondria should be immediately used, do not freeze them. Keep samples on ice during the enzymatic activity assays.

### Measurement of mitochondrial respiratory chain enzymatic activities

The general concept behind measurement of the mitochondrial respiratory chain activities is to follow each enzymatic reaction through time. The enzyme kinetics is assessed via spectrophotometry by following variations in the UV-VIS absorbance of colorimetric substrates or reaction products. To date, most of UV-VIS plate readers and standard cuvette spectrophotometer can be set up to register absorbance during a time period.

If a 96-well plate is used, as described in this protocol, we suggest following kinetic activity by registering absorbance values for 2 min, every 20 or 30 s, depending on the number of samples measured and reading speed of the plate reader at the desired wavelength. A plate reader is highly advised to measure multiple samples at the same time and to save reagents, as assays are carried out in 200 μL of reaction volume. If a standard cuvette spectrophotometer is used, adjust the reaction volume as needed.

***Note:*** the measurement of mitochondrial respiratory chain enzymatic activities should be carried out at 30°C.

***Note:*** allow reaction buffers to warm up at 20°C–25°C before the assays.

***Note:*** enzyme activities should be measured at least in triplicate plus at least one replicate reaction in presence of a selective inhibitor to calculate specific activity.

#### Measurement of complex I (NADH:ubiquinone oxidoreductase) activity

**Timing: 0.5–1 h**

Complex I is a NADH:ubiquinone oxidoreductase. Complex I is a starting point of the mitochondrial respiratory chain, the other being Complex II and other FADH_2_-dependent enzymes. It oxidizes NADH generated by the Krebs cycle in the mitochondrial matrix, passing two electrons through one flavin mononucleotide (FMN) and a series of iron-sulfur (Fe-S) clusters, to the electron carrier CoQ.

The activity of complex I is measured through a direct method, following the decrease in absorbance at λ=340 nm due to the oxidation of NADH, carried out by the enzyme (Estornell et al., 1993; Spinazzi et al., 2012).

14. For each replicate plus one (to ensure there is enough for all the samples), prepare the reaction mix as described in the following table:

**Table:** 

Reagent	Stock concentration	Volume (μL)	Final concentration
Potassium phosphate buffer pH 7.5	0.5 M	20	50 mM
BSA	50 mg/mL	12	3 mg/mL
KCN	10 mM	6	300 μM
NADH	5 mM	4	100 μM
H_2_O	–	144	–
Rotenone∗	1 mM	2	10 μM

15. Add 186 μL of the reaction mix into each well of a 96- well plate (see key resources table), using a multichannel pipette.
16. Add 10 μL of mitochondrial lysate from step 13 (1 μg/μL) and mix well.
17. Record baseline activity at λ=340 nm for 2 min at 30°C.
18. Add 4 μL of 4 mM CoQ_1_ (final concentration 80 μM) to each well to start the reaction. Make sure the substrate is mixed well in the reaction mix.
19. Promptly record the absorbance at λ=340 nm for 2 min at 30°C.
20. ∗To assess rotenone sensitive NADH-dehydrogenase activity (specific to mitochondrial complex I), perform parallel reactions in presence and absence of rotenone (complex I inhibitor).

**Figure 1:**
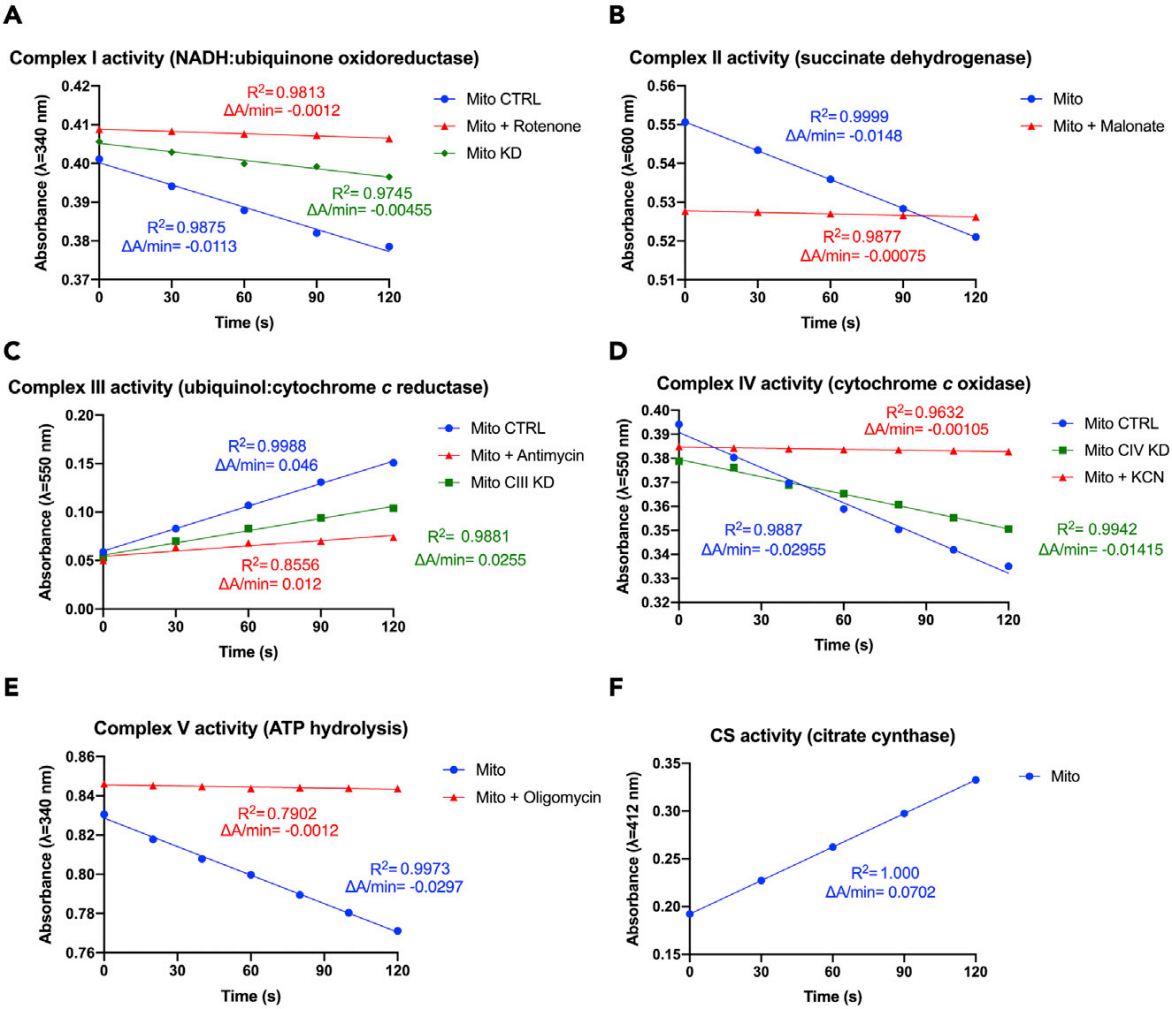
Expected outcomes for enzymatic activity assays (A) Complex I activity recordings of wild-type mitochondria in absence (Mito) or presence of the inhibitor (Mito + rotenone), plus recording of mitochondria from complex I subunit *ND-18* (*Ndufs4*) gene knockdown *D. melanogaster* individuals (Mito CI KD), (Foriel et al., 2018). (B) Complex II activity recordings of wild-type mitochondria in absence (Mito) or presence of the inhibitor (Mito + Malonate). (C) Complex III activity recording of wild-type mitochondria in absence (Mito) or presence of the inhibitor (Mito + Antimycin A) plus recording of mitochondria from complex III assembly factor *Bcs1* gene knockdown *D. melanogaster* individuals (Mito CIII KD), (Brischigliaro et al., 2021). (D) Complex IV activity recording of wild-type mitochondria in absence (Mito) or presence of the inhibitor (Mito + KCN) plus recording of mitochondria from complex IV assembly factor *Coa8* gene knockdown *D. melanogaster* individuals (Mito CIV KD), (Brischigliaro et al., 2019). (E) Complex V activity recordings of wild-type mitochondria in absence (Mito) or presence of the inhibitor (Mito + Oligomycin). (F) CS activity recordings of wild-type mitochondria (Mito).

***Note:*** expected results are reported in Figure 1A.

#### Measurement of complex II (succinate:coenzyme Q oxidoreductase) activity

**Timing: 0.5–1 h**

Complex II is a succinate:coenzyme Q oxidoreductase - succinate dehydrogenase - that oxidizes succinate to fumarate during the sixth step of the TCA cycle in the mitochondrial matrix. It couples the oxidation to the reduction of the electron carrier CoQ. The activity of complex II is measured through an indirect method following the decrease in absorbance at λ=600 nm due to the reduction of DCPIP. DCPIP is reduced by CoQ_1_ from the transfer of electrons from succinate to CoQ through the activity of complex II (Trounce et al., 1996; Spinazzi et al., 2012).

21. For each replicate plus one, prepare the reaction mix as described in the following table:

**Table:** 

Reagent	Stock concentration	Volume (μL)	Final concentration
Potassium phosphate buffer pH 7.5	0.5 M	10	25 mM
BSA	50 mg/mL	4	1 mg/mL
KCN	10 mM	6	300 μM
Succinate	400 mM	10	20 mM
DCPIP	500 μM	30	75 μM
H_2_O	–	136	–
Malonate∗	1 M	2	10 mM

22. Add 196 μL of the reaction mix into each well of a 96- well plate (see key resources table), using a multichannel pipette.
23. Add 2 μL of mitochondrial lysate from step 13 (1 μg/μL) and mix well.
24. Shake the plate for 5 s.
25. Pre-incubate the plate at 25°C for 10 min.
26. Record baseline activity at λ=600 nm for 2 min at 30°C.
27. Add 2 μL of 4 mM CoQ_1_ (final concentration 40 μM) to each well to start the reaction.
28. Shake the plate for 5 s, wait for 5 s.
29. Record absorbance at λ=600 nm for 2 min at 30°C.
30. ∗To subtract the contribution of unspecific reactions, perform parallel reactions in presence and absence of malonate (complex II inhibitor).

***Note:*** During complex II activity assay, baseline activity reflects succinate dehydrogenase (SDH) activity, as complex II is able to reduce DCPIP when CoQ_1_ is not present, although at slower rate.

***Note:*** Expected results are reported in Figure 1B.

#### Measurement of complex III (ubiquinol:cytochrome *c* oxidoreductase) activity

**Timing: 0.5–1 h**

Complex III, also known as cytochrome *bc1* complex, is a ubiquinol:cytochrome *c* oxidoreductase that catalyzes the electron transfer from ubiquinol (CoQ) to the second mobile electron carrier of the MRC, cytochrome *c*. The activity of complex III is measured through a direct method, following the increase in absorbance at λ=550 nm due to the reduction of cytochrome *c*, carried out by the enzyme (Trounce et al., 1996; Spinazzi et al., 2012).

31. For each replicate plus one, prepare the reaction mix as described in the following table:

**Table:** 

Reagent	Stock concentration	Volume (μL)	Final concentration
Potassium phosphate buffer pH 7.5	0.5 M	10	25 mM
EDTA pH 7.5	5 mM	4	100 μM
KCN	10 mM	10	500 μM
Tween-20	2.5%	2	0.025%
Cyt *c*_*ox*_	1 mM	15	75 μM
H_2_O	–	155	–
Antimycin A∗	1 mg/mL	2	10 μg/mL

32. Add 196 μL of the reaction mix into each well of a 96- well plate (see key resources table), using a multichannel pipette.
33. Add 2 μL of mitochondrial lysate from step 13 (1 μg/μL) and mix well.
34. Shake the plate for 5 s, wait for 5 s.
35. Record baseline activity at λ=550 nm within 2 min at 30°C.
36. Add 2 μL of 10 mM decylubiquinol (final concentration 100 μM) to each well to start the reaction.
37. Shake the plate for 5 s, wait for 5 s.
38. Record absorbance at λ=550 nm for 2 min at 30°C.
39. ∗To subtract the contribution of possible unspecific reactions, perform parallel reactions in presence and absence of antimycin A (complex III inhibitor).

***Note:*** It is crucial that the cytochrome *c* used for this assay is the product with Merk catalog number C7752, or similar (prepared with acetic acid without using TCA). Other kinds of cytochrome *c* isolated using other methods result in very high rates of unspecific cytochrome c reduction, greatly interfering with the assay.

***Note:*** expected results are reported in Figure 1C.

#### Measurement of complex IV (cytochrome *c* oxidase) activity

**Timing: 0.5–1 h**

Complex IV, or cytochrome *c* oxidase, is the terminal enzyme of the ETC. It catalyzes the oxidation of cytochrome *c* and the reduction of molecular oxygen (O_2_) to water (H_2_O). The activity of complex IV is measured through a direct method, following the decrease in absorbance at λ=550 nm due to the oxidation of reduced cytochrome *c*, carried out by the enzyme (Wharton and Tzagoloff, 1967; Trounce et al., 1996; Spinazzi et al., 2012).

40. For each replicate plus one, prepare the reaction mix as described in the following table:

**Table:** 

Reagent	Stock concentration	Volume (μL)	Final concentration
Potassium phosphate buffer pH 7.0	0.1 M	100	50 mM
Cyt *c*_*red*_ (1 mM)	1 mM	15	75 μM
H_2_O	–	83	–
KCN∗	10 mM	10	500 μM

41. Add 198 μL of the reaction mix into each well of a 96- well plate (see key resources table), using a multichannel pipette.
42. Shake the plate for 5 s, wait for 5 s.
43. Record baseline activity at λ=550 nm within 2 min at 30°C.
44. Add 2 μL of mitochondrial lysate from step 13 (1 μg/μL), which will start the reaction. Mix well.
45. Shake the plate for 5 s, wait for 5 s.
46. Record absorbance at λ=550 nm for 2 min at 30°C.
47. ∗To subtract the possible contribution of unspecific reactions, perform parallel reactions in presence and absence of KCN (complex IV inhibitor).

***Note:*** expected results are reported in Figure 1D.

#### Measurement of complex V (ATP synthase) activity

**Timing: 0.5–1 h**

Complex V is a F-type ATP synthase. It is a rotary enzyme that couples proton translocation, driven by the proton motive force (PMF) generated by proton translocation at the level of complexes I, III and IV, with ATP synthesis. It is also known as F_O_F_1_- ATP synthase.

***Note:*** currently, no kinetic spectrophotometry-based methods are available for directly assessing ATP synthesis by ATP synthase. Therefore, the most commonly used methods are based on the evaluation of the reverse activity (ATP hydrolysis) of the enzyme, which have been proven useful to determine Complex V activity in isolated mitochondrial samples. A luminometric assay for measuring mitochondrial ATP synthesis was described before (Vives-Bauza et al., 2007).

This activity is measured through an indirect method by linking its ATPase activity to NADH oxidation. Firstly, in presence of ADP (resulting from hydrolytic activity of ATP synthase), PEP is converted to pyruvate + ATP by pyruvate kinase. Then, pyruvate is converted to lactate by lactate dehydrogenase, through the oxidation of NADH, which is what this assay determines by measuring the decrease in absorbance at λ=340 nm.

48. For each replicate plus one, prepare the reaction mix as described in the following table:

**Table:** 

Reagent	Stock concentration	Volume (μL)	Final concentration
HEPES/Mg Buffer pH=8.0	100 mM / 10 mM	100	50 mM / 5 mM
NADH	5 mM	12	0.3 mM
Phosphoenolpyruvate	50 mM	10	2.5 mM
Pyruvate kinase	10 mg/mL	1	50 mg/mL
Lactate dehydrogenase	5 mg/mL	2	50 mg/mL
Antimycin A	0.2 mg/mL	2	2 mg/mL
H2O	–	50	–
Oligomycin∗	0.2 mg/mL	2	2 μg/mL

49. Add 177 μL of the reaction mix into each well of a 96 well plate (see key resources table), using a multichannel pipette.
50. Add 3 μL of mitochondrial lysate from step 13 (1 μg/μL) and mix well.
51. Pre-incubate at 25°C for 5 min.
52. Record baseline activity at λ=340 nm within 2 min at 30°C.
53. Add 20 μL of 25 mM ATP pH 7.0 (final concentration 2.5 mM) to each well to start the reaction.
54. Record absorbance at λ=340 nm for 2 min at 30°C.
55. ∗To subtract the possible contribution of unspecific reactions, perform parallel reactions in presence and absence of oligomycin (complex V inhibitor).

***Note:*** expected results are reported in Figure 1E.

#### Measurement of citrate synthase activity

**Timing: 0.5–1 h**

Citrate synthase catalyzes the condensation of acetyl-CoA and oxaloacetate to form citrate during the first step of the Krebs cycle in the mitochondrial matrix. The activity of citrate synthase is often used as a reliable method of normalization of MRC activity assays. The activity of citrate synthase is measured through an indirect method, following the formation of a yellow-colored thionitrobenzoate (TNB) derivative of DTNB by measuring the changes in absorbance at λ=412 nm. DTNB spontaneously forms TNB by reacting with sulfhydryl groups of free coenzyme A resulting from citrate synthase activity (Srere, 1969; Trounce et al., 1996; Spinazzi et al., 2012).

56. For each replicate plus one, prepare the reaction mix as described in the following table:

**Table:** 

Reagent	Stock concentration	Volume (μL)	Final concentration
Tris-HCl pH 8.0 + 0.2% Triton X-100	0.2 M	100	100 mM
DTNB in Tris-HCl pH 8.0	1 mM	20	100 μM
Acetyl-CoA (10 mM)	10 mM	6	300 μM
H_2_O	–	62	–

57. Add 188 μL of the reaction mix into each well of a 96 well plate (see key resources table), using a multichannel pipette.
58. Add 2 μL of mitochondrial lysate from step 13 (1 μg/μL) and mix well.
59. Record baseline activity at λ=412 nm within 2 min at 30°C.
60. Add 10 μL of 10 mM oxaloacetate (final concentration 500 μM) to each well to start the reaction.
61. Record absorbance at λ=412 nm for 2 min at 30°C.

***Note:*** expected results are reported in Figure 1F.

## Expected outcomes

This protocol provides detailed methodology for measuring the activity of each individual MRC enzymatic complex and the F_O_F_1_-ATPase in isolated mitochondria from *Drosophila* tissues. The data collected with this procedure describe the kinetic enzyme activity of the MRC, which in turn provides quantitative data about the amount and/or functionality of the individual complexes in biological samples.

This protocol records absorbance variations through time (ΔAbsorbance/minute - ΔA/min), which are used to calculate the enzymatic activity (Figure 1). The ΔA/min is calculated by subtracting the absorbance value at the end of the reaction to the absorbance value at the beginning of the reaction. Data collected can be plotted in Microsoft Excel and linear regression should be performed to evaluate R^2^ values of the reactions. Generally, R^2^>0.95 is considered a good result (Figure 1). For R^2^<0.95 we suggest repeating the measurement (see troubleshooting section), as this suggests non-linear reaction and unreliable assessment of enzymatic activity. Low R^2^ values for reactions in presence of inhibitors should not be considered problematic, as background variations in absorbance could contribute when low activities are recorded (Figure 1).

Specific activity is assessed by subtracting ΔA/min values recorded in presence of specific inhibitors of MRC complexes to ΔA/min values recorded in absence of the inhibitor. Generally, good mitochondrial preparations have very low enzymatic activities in presence of the inhibitor (>85%–90% of activity inhibited), (Figure 1).

### Enzyme activity calculations

To obtain quantitative enzymatic specific activity (SA) values, expressed as amount of substrate (nmol) converted per time unit (min) per mass unit (mg) of mitochondrial protein (nmol min^-1^ mg^-1^), use the following formula:

$$\frac{\left(\frac{\text{ΔA}}{\text{min}}\times \text{ mL}\;\text{of}\;\text{reaction}\right)}{\left(\text{ε }\times \text{ mL}\;\text{of}\;\text{sample }\times \text{ mg}/\text{mL}\;\text{of}\;\text{sample }\;\text{protein}\right)}$$

The molar extinction coefficients (ε) at the specific wavelengths (λ) of the different molecules for which the absorbance is measured in each assay can be found in the following table:

**Table:** 

Assay	Reagent	λ (nm)	ε (mM^-1^ cm^-1^)
CI	NADH	340	6.22
CII	DCPIP	600	19.1
CIII	Cyt *c*	550	18.5
CIV	Cyt *c*	550	18.5
CV	NADH	340	6.22
CS	TNB	412	13.8

For example, the calculations for complex I activities reported in Figure 1A are:

• Mito control: $\frac{\left(0.0113\frac{\text{ΔA}}{\text{min}}\times 0.2\times 1000\right)}{\left(6.22{\text{ mM}}^{-1}\times \;0.01\;\text{mL }\times \;1\;\text{mg}/\text{mL}\right)}=36.33\;\text{nmol}{\text{ min}}^{-1}{\text{ mg}}^{-1}$.
• Mito rotenone: $\frac{\left(0.0012\frac{\text{ΔA}}{\text{min}}\times 0.2\times 1000\right)}{\left(6.22{\text{mM}}^{-1}\times \;0.01\;\text{ mL }\times \;1\;\text{mg}/\text{mL}\right)}=3.85\;\text{nmol }{\text{min}}^{-1}{\text{ mg}}^{-1}$.
• Mito KD: $\frac{\left(0.00455\frac{\text{ΔA}}{\text{min}}\times 0.2\times 1000\right)}{\left(6.22{\text{mM}}^{-1}\times \;0.01\;\text{mL }\times \;1\;\text{mg}/\text{mL}\right)}=14.63\;\text{ nmol }{\text{min}}^{-1}{\text{ mg}}^{-1}$.

## Limitations

This method relies on the availability of enough amounts of tissue. For instance, if small larvae (e.g., 1^st^ or 2^nd^ instar larvae) and thoraces are being used, many individuals will be needed. Furthermore, dissection could be time consuming. In case of a complex experimental setup (e.g., many experimental conditions or genotypes) we suggest to prepare and store mitochondrial preparations before proceeding with the enzyme activity assays.

## Troubleshooting

### Problem 1

Low or no enzymatic activity observed for different MRC complexes.

### Potential solution

Samples may be degraded, make sure you perform all the procedures on ice using ice-cold buffers, make sure samples are store at -80 for long periods of time and avoid multiple freeze and thaw cycles of the samples. Sample amount may be too low, try to increase sample amount until you observe linear reactions significantly higher than the background.

### Problem 2

Non-linear reactions or plateau phase observed.

### Potential solution

Excessive amount of sample used for the activity assay. Reduce the amount of sample.

### Problem 3

Activity observed during baseline recording.

### Potential solution

Contamination of buffers or reagents. Discard old reagents and prepare fresh solutions.

### Problem 4

Low complex I activity.

### Potential solution

Oxidation of NADH. Prepare fresh NADH solution. NADH solution should be colorless or light yellow. Yellow color indicates oxidation of NADH.

### Problem 5

High non-specific (rotenone-insensitive) complex I activity.

### Potential solution

Contamination with reticular or cytosolic NADH dehydrogenases. Thoroughly wash the mitochondrial pellets twice by resuspending in the appropriate amount of buffer. Make sure that centrifuge speed is correctly set.

### Problem 6

Low complex II activity.

### Potential solution

Pre-incubation step was skipped. Pre-incubate mitochondria with assay solution at least 10 min before measuring the activity.

### Problem 7

Low or no complex III activity.

### Potential solution

Carry over of potassium borohydride used for reduction of decylubiquinone. Avoid excess potassium borohydride. Make sure the decylubiquinol solution stops bubbling after addition of HCl. If bubbling does not stop after addition of 2–3 HCl aliquots, repeat centrifugation step and carefully avoid touching the bottom of the tube.

### Problem 8

Low or no complex IV activity.

### Potential solution

Carry over of sodium dithionite used for reduction of cytochrome *c*. Avoid excess dithionite that can counteract complex IV activity.

### Problem 9

Low or no complex V activity.

### Potential solution

Complex V activity assay relies on pyruvate kinase and lactate dehydrogenase activities. Store enzymes at 2°C–8°C.

## Resource availability

### Lead contact

Further information and requests for resources and reagents should be directed to and will be fulfilled by the lead contact, Carlo Viscomi (carlo.viscomi@unipd.it).

### Materials availability

This study did not generate new unique reagents.

## Data Availability

This study did not generate/analyze datasets/code.
